# Oral Hygiene

**Published:** 1898-04

**Authors:** Eugene S. Talbot

**Affiliations:** Chicago.


ARTICLE VII.

### ORAL HYGIENE.

BY EUGENE S. TALBOT, M. D., D. D. S., CHICAGO.

**Read before XIIth International Medical Congress at Moscow, Russia, August 18-26, 1897.**

That the mouth and teeth should be kept absolutely clean and free from deposits, every practitioner will admit is but an axiomatic statement. Neglect in this direction entails degeneration of the teeth and soft parts of the mouth, which in turn ultimately affects the general health. Many dentists and most patients are unmindful of the necessity of keeping the gums and mucous membrane healthy. Frequently, despite strict injunctions are given, the patient returns with the mouth in the most unhygienic condition. The patient has gradually lost ambition and allowed the teeth and mouth to fall far beneath ideal prophylaxis.

As a profession dentists are apt to congratulate themselves upon the rapid advancement made in the specialty. forgetting that bacteriologic research demonstrates a fact which physicians and dentists have suspected, that the mouth is the culture medium for germs which, when opportunity offers, play their part in producing many diseases which flesh is heir to.

Galippe, Black and Miller, after special microscopic study of fluids and excretions of the mouth, have shown that this organ is a very fruitful field for the propagation of bacteria. Miller says, "Within the last few years I have isolated more than too different kinds of bacteria from the juices and deposits in the mouth. Physiologists and chemists have also shown that foods, especially starch and sugar, taken into the mouth, on coming in contact with the saliva, chemical changes immediately take place, as a result of which lactic acid is produced. Miller has found that lactic acid is the source from which decay of the teeth results.

It would be strange if some of the numerous bacteria, under favorable environment, should not produce diseases of the mouth, alimentary canal and general system.

Micro-organisms are more destructive in the mouths of persons whose vitality is below par than those who are normal. People who have been ill for any length of time soon realize that the teeth are giving way, that pus germs infect the mouth and that the soft tissues are very unhealthy. A class coming for treatment, whose mouths are exceedingly prone to disease, is that of the degenerates. It is almost impossible at present for the majority of dentists to distinguish this class from the normal, yet they exist in dental practice in large numbers.

The mouths of this class are in an unhealthy condition; the teeth irregular and often badly decayed, gums and mucous membrane swollen, with pyorrheal oozing from gums and teeth. Frequently ulcers occur at different localities, and saliva flows freely from enlarged glands.

Degeneracy is stamped upon their faces. The bones and mucous membrane of the nose are often hypertrophien. In such cases adenoid vegetation, together with other obstructing factors, prevents nose-breathing, and mouth-breathing results. Necessarily many germs otherwise absent are taken into the mouth, which afterwards infect the lungs and alimentary canal. These types of mouth are observed in their most exaggerated forms among the imbeciles, dements and stuporous lunatics. Local treatment of such patients would be fallacy.

I lay stress upon this class of cases, because of the lack of literature on the subject and the difficulty of impressing the patient with the importance of the care of the mouth. Such persons, even if otherwise fairly well balanced, are too unstable and have not sufficient continuous will power to carry out successfully instructions of the operator. These patients, including those who are afflicted with disease, require constitutional treatment to restore the tonicity of structure, after which local treatment proves successful.

I would not, however, for one moment be thought to discourage the attempt to keep the mouth in hygienic condition, especially in cases of invalids and small children, who must receive the greatest care from nurse or parent, As absence of general tonicity will prevent a local restoration to health, in like manner will lack of local cleanliness cause systemic disturbances. Besides illness, worry, anxiety, trouble will produce malnutrition and tissue degeneration.

It is not necessary to discuss the various bacteria and their action upon the fluids and tissues of the mouth, since this has been thoroughly brought before the profession by Miller. To treat the subject of this paper in a proper manner it is necessary to speak only of a few forms of bacteria which, if destroyed, the other kinds will succumb.

Two of the most marked forms of degeneration of tissue as the result of uncleanliness are tooth decay and gum inflammation, resulting in pyorrhea. Pasteur has remarked, "that whenever and wherever there is decomposition of organic matter—whether it be the case of an herb or an oak, of a worm or whale—the work is exclusively done by infinitely small organisms." Therefore I commend the following propositions: If the mouth and teeth were kept absolutely clean, so that chemical changes could not take place, the teeth would certainly never decay, no matter how imperfectly the enamel might develop. If the gums be stimulated in a proper manner, and kept in a healthy condition, they are decidedly less liable to become infected with pyorrhea.

Bearing these two propositions in mind and striving to attain the results, the mouth will become comparatively healthy. How then shall we accomplish the desired object? Use of proper tooth-brush, mouth wash and a knowledge of their use, is the indication. The ordinary toothbrush falls far short of what is required for proper care of the teeth. Most have soft bristles, or else they become soft, for the reason that they are sometimes kept in a closed vessel or used so often that they are not allowed to dry. A soft-bristle brush should never be used, only a medium or hard one. The size of the brush is also to be taken into consideration. Most of them are too large and on that account it is difficult to get beyond the molars. The shape of the brush is also to be borne in mind. Dr. M. L. Rhein, of New York, has taught the evolution of the toothbrush. The curved handle and small end which contain the bristles are certainly a great improvement over straight handles or those bent in the opposite direction from the bristles. The tuft and concavity of the bristles (so far as they go) are an improvement over bristles which are at right angles to the handle. This allows the posterior portion of the molars as well as the spaces between the teeth to be reached when the brush is properly used.

With the proper use of such a brush three times a day, and floss silk twice a day, any person of ordinary intelligence, after being instructed, can keep the teeth clean. There can be no question but that if the gums be well stimulated and an antiseptic wash used, pyorrhea will never appear. Pyorrhea is due either to simple or chronic inflammation of the gums, no matter whether the cause be local or constitutional. Both simple and chronic inflammation may be reduced, first by blood-letting, to relieve the congestion, and second, by stimulation of all the parts. This, however, cannot be accomplished with the toothbrushes now in use. The bristles are not only too soft, but they are so close together that when the brush is applied to the tooth, those bristles which come in contact with the teeth prevent the others reaching the gums.

I have given this subject considerable study, the result of which is the following brush, which for the past two years I have had my patients use, and with the greatest satisfaction. It is made similar to the prophylactic, only smaller in every way. Spaces are left at every two rows, so that the teeth will pass in between them and the bristles reach the gums. Between the tuft upon the end and the body of the brush quite a space is left, so that the front teeth, upper and lower, will go between and the tuft reach the gums above, below and posterior to the incisor teeth. Inflammation of the gums starts most frequently at these localities first and is usually in its most severe form, since with the ordinary toothbrush it is more difficult to reach these parts.

It is not an easy matter to find a mouth-wash that will destroy bacteria, and at the same time not become injurious to the mucous membrane. Miller, after repeated experiments, says, "An examination of the above results will soon convince us that there are very few substances at present in the dental materia medica which are available for disinfecting the human mouth." After experimenting with twenty-six different drugs, it was found that with one exception, not one of these drugs would destroy bacteria in less than five minutes, most of them requiring over eight minutes. Out of all the drugs which are used daily, trichlorid of iodin destroyed bacteria in one and one-fourth minutes, saccharin in three-fourths to one minute, benzoic acid in two to two and one-half minutes, salicylic acid in three-fourths to one minute. Of these benzoic acid seems to be the only drug from which a suitable mouth-wash can be made. Listerine and thymol have been favorite mouth-washes in America, but lately a new preparation has become very popular, namely Borolyptol. It is composed of five per cent. aceto-boro-glycerid O., two per cent. formaldehyd, in combination with the antiseptic constituents of pinus, pumilio, eucalyptus, myrrh, storax, benzoin. Experiments made by able chemists have shown that it is fully as strong and will destroy bacteria as readily as 1-1,000 bichlorid of mercury.

I have used borolyptol in my practice for the past year with success, especially in cases of pyorrhea alveolaris, without injury to the mucous membrane. Miller says, "that trichlorid of iodin is decidedly superior to the bichlorid; it is furthermore far less disagreeable than the latter, in fact, not at all disagreeable, it has, however, most unfortunately, an acid reaction, and is therefore not suitable for daily use as a mouth-wash." Nevertheless, in cases of pyorrhea alveolaris after the deposits are removed, I know of nothing better than the officinal preparation of iodin, saturating the gums and pockets throughout three times a week. It acts not only as an astringent, but it prevents putrefactive changes, decomposes sulphurated and phosphorated compounds; it therefore acts as a powerful astringent and will destroy most forms of bacteria. These two drugs, with a faithful application of the toothbrush, are all that is necessary to keep the mouth in a healthy condition. By this method the worst forms of diseased gums and inflamed mucous membrane can be successfully treated.

In regard to tooth-powder, I feel confident that many dentists make a mistake in recommending nothing but soft substances. One writer upon the subject of oral hygiene says, in speaking of a tooth-powder, "It should contain a scouring or polishing agent of the nature of a minutely-divided powder, such as precipitated chalk." I would like to ask how much scouring and polishing can be done with chalk? There is no objection to using the chalk for bulk, but to scour and polish the teeth I prescribe equal parts precipitated chalk and powdered cuttle-fish, carmin to color, orris root and fine sugar to sweeten. This mixture, when rubbed up and put through a No. 60 sieve, makes an excellent powder which may be used two or three times a week.—*Dental Digest.*

